# GROWTH FACTORS AND COX2 IN WOUND HEALING: AN EXPERIMENTAL STUDY WITH EHRLICH TUMORS

**DOI:** 10.1590/0102-6720201600040003

**Published:** 2016

**Authors:** Flávio L. L. SALGADO, Ricardo ARTIGIANI-NETO, Gaspar de Jesus LOPES-FILHO

**Affiliations:** 1 Postgraduate Program in Interdisciplinary Surgical Science, Paulista School of Medicine, Federal University of São Paulo - UNIFESP, São Paulo, SP, Brazil

**Keywords:** Carcinoma, Ehrlich Tumor, Wound healing, Cyclooxygenase 2. Vascular endothelial growth factor receptor-1, Fibroblast growth factor 2

## Abstract

**Background:**

Healing is an innate biological phenomenon, and carcinogenesis acquired, but with common humoral and cellular elements. Carcinogenesis interferes negatively in healing.

**Aim:**

To evaluate the histological changes in laparotomy scars of healthy Balb/c mice and with an Ehrlich tumor in its various forms of presentation.

**Methods:**

Fifty-four mice were divided into three groups of 18 animals. First group was the control; the second had Ehrlich tumor with ascites; and the third had the subcutaneous form of this tumor. Seven days after tumor inoculation, all 54 mice were submitted to laparotomy. All of the animals in the experiment were operated on again on 7^th^ day after surgery, with resection of the scar and subsequent euthanasia of the animal. The scars were sent for histological assessment using immunohistochemical techniques to evaluate Cox-2 (cyclooxygenase 2), VEGF (vascular endothelial growth factor) and FGF (fibroblast growth factor). Semi-quantitatively analysis was done in the laparotomy scars and in the abdominal walls far away from the site of the operation.

**Results:**

Assessing the weight of the animals, the correct inoculation of the tumor and weight gain in the group with tumoral ascites was observed. The histological studies showed that groups with the tumor showed a statistically significant higher presence of Cox-2 compared to the control. In the Cox-2 analysis of the abdominal wall, the ascites group showed the most significant difference. VEGF did not present any significant differences between the three groups, regardless of the site. The FGF showed a significant increase in animals with the tumor.

**Conclusion:**

Histological findings in both laparotomy scar and the abdominal wall showed that with Ehrlich's neoplasia there was an exacerbated inflammatory response, translated by more intense expression of Cox-2 and greater fibroblast proliferation, translated by more intense expression of FGF, that is, it stimulated both the immediate inflammatory reactions, observed with Cox-2 reactions, and late scarring by fibroblasts and FGF.

## RESUMO

**Racional:**

A cicatrização é fenômeno biológico inato, e a carcinogênese adquirido, mas com elementos humorais e celulares comuns. A carcinogênese interfere de forma negativa na cicatrização.

**Objetivo:**

Avaliar as modificações histológicas nas cicatrizes laparotômicas de camundongos Balb/c sadios como controles, e com a neoplasia de Ehrlich, em suas diferentes formas de apresentação.

**Métodos:**

Foram utilizados 54 camundongos, divididos em três grupos de 18 animais cada um. O primeiro era controle; o segundo com a neoplasia de Ehrlich em sua forma ascítica; e o terceiro na forma subcutânea. Sete dias após a inoculação do tumor, todos os 54 camundongos foram submetidos à laparotomia e reoperados no sétimo dia de pós-operatório, com ressecção da cicatriz e posterior eutanásia. As cicatrizes foram encaminhadas para estudo histológico com técnicas imunoistoquímicas para avaliar Cox-2 (ciclo-oxigenase 2), VEGF (fator de crescimento do endotélio vascular) e FGF (fator de crescimento dos fibroblastos) e analisadas de forma semiquantitativana tanto na cicatriz laparotômica como na parede abdominal mais distante do local operado.

**Resultados:**

Avaliando o peso, observou-se a correta inoculação do tumor e o aumento de peso no grupo com a neoplasia na modalidade ascítica. Os estudos histológicos mostraram que os grupos com a neoplasia apresentaram maior presença da Cox-2 em relação ao controle, estatisticamente significante. No estudo da Cox-2 da parede abdominal foi o local em que o grupo ascítico apresentou a diferença mais expressiva. O VEGF não apresentou diferenças significantes entre os três grupos, independentemente do local estudado. O FGF teve aumento significante nos animais com neoplasia.

**Conclusão:**

Os achados histológicos encontrados tanto na cicatriz das laparotomias quanto na parede abdominal mostraram que com a neoplasia de Ehrlich houve resposta inflamatória exacerbada, traduzida por expressão mais intensa da Cox-2 e maior proliferação fibroblástica, traduzida por expressão mais intensa do FGF, ou seja, estimulou tanto as reações inflamatórias imediatas, observadas nas reações da Cox-2, como nas cicatriciais tardias com os fibroblastos e o FGF.

## INTRODUCTION

Healing is defined as the organism's capacity to repair tissues after inflammation^3^ ^,^ ^4^ ^,^ ^13^. Wound healing is a complex repairing phenomenon, involving inflammatory reaction, a proliferative process and then tissue remodeling^22^. Complete regeneration, which could be defined as the formation of a tissue with identical structure and function to the original, is uncommon^22^. Rather, cicatrization or healing is a process in which the integrity of the tissue is obtained in a similar manner, with small modifications depending on the site, the tissue of the wound, and the agent causing it^3^ ^,^ ^4^ ^,^ ^13^.

Studies described five stages of healing, beginning with the acute inflammatory phase, then cellular proliferation, formation of conjunctive tissue, contraction and final remodeling of the wound. These phases are mixed and not individualized; they occur together and overlap each other. Some authors cite four phases: cleaning, granulation tissue, re-epithelialization and retraction^8^ ^,^ ^10^ ^,^ ^22^.

Granulation tissue is the new tissue that grows and fills the defect from the wound. It includes the proliferation of fibroblasts and capillaries. The capillaries originate from endothelial cells. Angiogenesis appears to be similar in the granulation of wounds and the creation of stroma for neoplastic cells in the growth of malignant tumors^2^.

In the healing process various mediators are synthesized in the damaged region, which modulate the inflammatory cascade in the early stage of injury repair and the formation of a permanent scar^7^. Of these, Cox 2 and pro-inflammatory cytokines, such as interleukins, growth factors and tumor necrosis factor, are fundamental for cell proliferation and synthesis of the extracellular matrix. Cytokines acting on injuries and malignant tumors can reduce the expression of p53, a regulator of cell replication^18^ ^,^ ^28^ ^,^ ^31^.

The reduced VEGF decreases expression and protein synthesis and the deposition of collagen, worsening healing. Research shows that the introduction of VEGF in the tissue of animals causes a local increase in vascularization^29^ ^,^ ^30^. Several solid tumor studies have demonstrated the importance of VEGF in the angiogenesis of tumors^1^. The formation of the extracellular matrix and collagen deposition depend on the inflammatory reaction^11^. In the stage of cellular proliferation and connective tissue formation, wound healing is similar in some respects to that observed in some tumors.

The objective of this study was to compare the histological changes in laparotomy scars in healthy mice and those with Ehrlich tumors.

## METHODS

The experiment followed the ethical principles for animal experiments and animal care and pain control, and the local legislation. The project was approved by the university's Ethics Committee (protocol number 2009/09).

### Study design and animals

This is an experimental, controlled study with female Balb/c mice. All animals were from the same university bioterium, weighing 18-22 g, aged nine weeks old. Before the experiment, the animals spent seven days in the laboratory to acclimatize to the environment. The space had controlled temperature (between 20-24 degrees Celsius) and humidity (at 50-60%), with dark/light cycles of 12 h, and mice had free access to standardized dry pellet diet and water. They were kept in individual cages and were evaluated daily and weighed four times during the experiment. Operated animals were kept in a different room of the rats awaiting surgery. The animals were fastened for food for 6 h and for water for 1 h before surgery. A pilot study with six animals was performed before the experiment for the standardization of anesthetic, surgical and histopathologic analysis techniques.

Three groups were studied with 18 animals each: control group (C); group with introduction of Ehrlich tumor cell into the peritoneum^6^ ^,^ ^9^ ^,^ ^12^ ^,^ ^26^ (A); and group where the same cell was introduced into the subcutaneous tissue of the left thigh (D). The introduction of the malignant cell in the two proposed models was performed according to an original work developed in 1896 and published in 1906 by Paul Ehrlich^9^ and followed by other research models^6^ ^,^ ^12^ ^,^ ^26^. Seven days after the introduction of the tumor cell or saline buffer acid, all 54 animals were subjected to median laparotomy. A week after surgery, the animals were operated once again with resection of the anterior abdominal wall tissue, including the surgical scar. This tissue block was sent for histological study.

### Neoplastic cells inoculation

Neoplastic cells with 92% of cell viability (counted in Neubauer chamber) were provided by the Veterinarian School of the University. The solution (0.4 ml) was injected into the peritoneum (in the the lower left quadrant of the abdomen) or into the subcutaneous layer of the left thigh (hind leg) of the mice (lateral external regional). 

### Surgical procedure, postoperative care and euthanasia

Before anesthesia, rats were sedated with atropine (0.04 mg/kg or 0.0012 mg for an animal weighing 30 g). Xylazine (50 mg/kg) and ketamine (10 mg/kg) were used for anesthesia. Drugs were administered intramuscularly in the right thigh of the mice. 

All animals underwent laparotomy, with a median incision of 2.5 cm. The abdominal contents were inspected in all animals, and the ascites liquid observed in the group that received neoplastic cells in the peritoneum was not eliminated. Two surgeons operated using esterile technique. The abdominal wall was closed on two planes with four stiches of Mononylon Ethicon 5-0 suture, 0.6 cm apart from each other approximately. The first plane involved the peritoneum wall, aponeurosis and muscles; and the second, the subcutaneous tissue and skin.

Animals were put back in their cages 30 minutes after surgery. Tramadol hydrochloride (1 mg/kg, each 12 hours) was used as analgesic for three days after surgery, added to the animals' water^5^. The surgical wound was kept exposed, with no dressings. 

Seven days after the first surgery, animals were euthanized with quadruplicated dosages of the drugs used in anesthesia. The surgical scar and tissues around it were removed en bloc for histological analysis, measuring approximately 3 cm in extension, from the xiphoid process. 

### Histological analysis

The tissue removed from the abdominal wall and the surgical scar of all animals was histologically analyzed. They were preserved in Carnoy solution for 12 h and then absolute alcohol (95%), and then submitted to dehydration, diaphanization with xylene and paraffin embedding. The slides were mounted with 5-micra thick sections, submitted to staining with hematoxylin and eosin (HE) and Masson's trichrome for scar identification. Immunohistochemistry study was performed for the detection of cyclooxygenase 2 (Cox-2 SP21, M3214 Spring clone, diluted at 1/250), vascular endothelial growth factor (VEGF C-1, SC7269 Santa Cruz clone, diluted at 1/100) and fibroblast growth factor (FGF 2, 147 Santa Cruz clone, diluted at 1/1500). 

Reactions were classified as light, moderate or intense, with scores varying from 1 to 3^14,16,20,^, in the scar and in the abdominal wall (for Cox 2 and VEGF) and in the inflammation infiltrate in scar tissue only for FGF.

### Statistical analysis

The Student's t-test was used to evaluate the weight of the animals within each group. ANOVA and Tukey's test were used for the analysis between the three groups. In the analysis of the histological parameters, the binomial test was used to test the differences between the independent sample proportions. BioEstat software, version 5.0, was used in statistical analysis, considering as significant p-values equal to or less than 0.05.

## RESULTS

**FIGURE 1:**
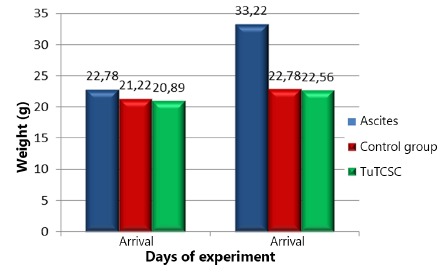
Weight on arrival and reoperation in all three groups

**FIGURE 2:**
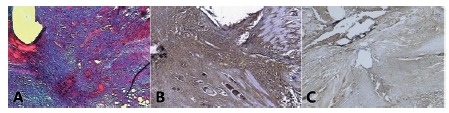
Surgical scar: A) Masson's trichrome, group D, 40x D; B) Cox-2, group A, 40x,+++; C) Cox-2, group A, 40x, ++

There were no deaths among the 54 animals during the experiment. There were no surgical or anesthetic complications. Animals in the group with ascites experienced weight gain of 10 g in average comparing baseline with the reoperation day (Figure 1). Weight in this group was significantly higher than in the other groups in the reoperation day (p<0.05). The H&E and Masson trichrome techniques were able to identify the surgical scar accurately (Figure 2A). In the Cox-2 evaluation within each group it was noted that in group A there was significant difference between the abdominal wall and the surgical wound data (p<0.05, Figures 2B and 2C). 

Comparing the three groups, there was a significant difference in the abdominal wall between groups A, D and C (p*<* 0.05), with higher expression in group A compared to D, and group D to C.

In the evaluation of the surgical scar, the tumor cell groups A and D showed no significant difference between them (p*>*0.05), but were higher when compared with group C (p*<* 0.05).

**FIGURE 3:**
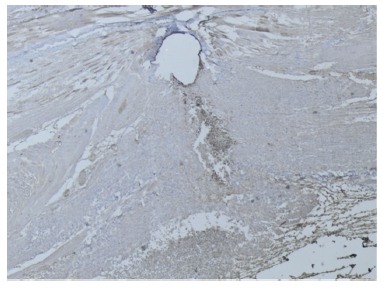
VEGF, group A, 40x,++

The analysis with VEGF showed no differences between the three groups in the surgical wound or abdominal wall (p*>* 0.05, Figure 3).

**FIGURE 4:**
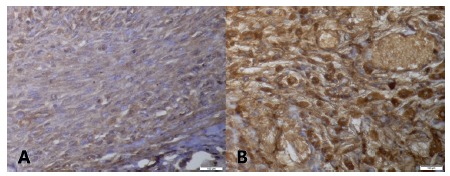
A) FGF, group D, 400x, ++; B) FGF, group A, 400x, +++

The FGF analysis showed that the scars of animals with tumors presented significant differences compared to the control (p*<*0.05). When compared, the groups with tumors showed no significant difference (p>0.05, Figures 4A and 4B). 

## DISCUSSION

The exclusive evaluation of the scar was impaired in group A owing to the invasion of the scar by neoplastic cells present in the ascites. This was the group where the tumor developed most rapidly with evident worsening of animals due to ascites and the symptoms induced by such. The abdominal wall distant from the scar was also assessed.

Cox-2 is an enzyme involved in the inflammatory response, also expressed in the cell division mechanisms of malignant tumors, where it plays an important role. It is usually not detected and increases in the carcinogenesis processes, when cytokines, stimuli and tumor growth factors exist, and acting in association with neoplastic tumor cells, can regulate the initial growth of these cells, their proliferation, invasion, migration, angiogenic phenomena and finally apoptosis and programmed death of the cells^19,21,27.^ In this study, the expression of Cox-2 was intense, at maximum level in most scars of animals with tumors from group A and group D. Expression was lower in group C. 

Angiogenesis consists in the formation of new vessels, driven by cellular factors such as macrophages, mast cells, platelets, fibroblasts and neoplastic cells that produce more than a dozen factors that interfere in the process, especially VEGF, FGF, and transforming growth factors β^15^ ^,^ ^25^. 

VEGF induces cell proliferation, cell migration, embryo development, cell differentiation, angiogenesis and malignant transformation. Angiogenesis is a fundamental biological correlate to malignancy and tumor progression. The capillaries originating from endothelial cells form and irrigate a stroma for the growth of neoplastic cells^24^. The differences in the inflammatory phase, as evaluated using Cox-2 in this experiment, were not repeated in the evaluation of VEGF in the scars. In the study of the entire abdominal wall, VEGF was found to be identical in all three groups. Infiltration of the abdominal wall by the tumor observed in group A also produced no differences in this finding.

FGF is a member of the family of growth factors that stimulate the proliferation of mesenchymal cells and epithelial cells. It stimulates the growth and maturation of fibroblasts, in conjunction with other cytokines. It was observed that all the conditions were created for the fibroblasts to have an increased expression in groups with tumors^17^ ^,^ ^23^. In this study, the evaluation of FGF is increased in the groups with tumors, that is, higher than in the control group.

The phases of healing, with humoral and vascular phenomena, should not be evaluated as closed and fractionated departments. The phenomena occur simultaneously, are self-regulating and interfere with each other. Separating cytokines from cells is impossible, because there is a fundamental interaction, not only for the healing process itself, but for its perfect understanding. In this study, the analysis of all the variables together demonstrates the possible interference of the neoplastic process with the operative scar.

The analysis of the histological findings using immunohistochemical methods, both in the laparotomy scars and in the abdominal wall, allows us to conclude that, in mice with Ehrlich tumors, there was exacerbated inflammatory response, reflected by the increased expression of Cox-2, as well as greater fibroblast proliferation, reflected by the increased expression of FGF. We did not detect changes in the vasculature due to lack of significant differences in the analysis of VEGF between groups. The exaggerated inflammatory response in the tumor groups explains the possible presence of a larger number of fibroblasts in this scar, producing increased secretion of collagen and fibrosis.

## CONCLUSION

Histological findings in both laparotomy scar and the abdominal wall showed that with Ehrlich's neoplasia there was an exacerbated inflammatory response, translated by more intense expression of Cox-2 and greater fibroblast proliferation, translated by more intense expression of FGF, that is, it stimulated both the immediate inflammatory reactions, observed with Cox-2 reactions, and late scarring by fibroblasts and FGF.
